# From Macroscopy to Ultrastructure: An Integrative Approach to Pulmonary Pathology

**DOI:** 10.3389/fmed.2022.859337

**Published:** 2022-03-16

**Authors:** Stijn E. Verleden, Peter Braubach, Christopher Werlein, Edith Plucinski, Mark P. Kuhnel, Annemiek Snoeckx, Haroun El Addouli, Tobias Welte, Axel Haverich, Florian P. Laenger, Sabine Dettmer, Patrick Pauwels, Veronique Verplancke, Paul E. Van Schil, Therese Lapperre, Johanna M. Kwakkel-Van-Erp, Maximilian Ackermann, Jeroen M. H. Hendriks, Danny Jonigk

**Affiliations:** ^1^Antwerp Surgical Training, Anatomy and Research Centre (ASTARC), Antwerp University, Antwerp, Belgium; ^2^Division of Pneumology, University Hospital Antwerp, Edegem, Belgium; ^3^Department of Thoracic and Vascular Surgery, University Hospital Antwerp, Edegem, Belgium; ^4^Member of the German Center for Lung Research (DZL), Biomedical Research in Endstage and Obstructive Lung Disease Hannover (BREATH), Hannover, Germany; ^5^Institute for Pathology, Hannover Medical School, Hannover, Germany; ^6^Division of Radiology, University Hospital Antwerp and University of Antwerp, Edegem, Belgium; ^7^Division of Pneumology, Hannover Medical School, Hannover, Germany; ^8^Division of Thoracic Surgery, Hannover Medical School, Hannover, Germany; ^9^Department of Radiology, Hannover Medical School, Hannover, Germany; ^10^Division of Pathology, University Hospital Antwerp, Edegem, Belgium; ^11^Laboratory of Experimental Medicine and Pediatrics (LEMP), Antwerp University, Antwerp, Belgium; ^12^Institute of Pathology and Department of Molecular Pathology, Helios University Clinic Wuppertal, University of Witten-Herdecke, Witten, Germany; ^13^Institute of Functional and Clinical Anatomy, University Medical Center of the Johannes Gutenberg University Mainz, Mainz, Germany

**Keywords:** microCT, lung, histology, lung disease, imaging

## Abstract

Pathology and radiology are complimentary tools, and their joint application is often crucial in obtaining an accurate diagnosis in non-neoplastic pulmonary diseases. However, both come with significant limitations of their own: Computed Tomography (CT) can only visualize larger structures due to its inherent–relatively–poor resolution, while (histo) pathology is often limited due to small sample size and sampling error and only allows for a 2D investigation. An innovative approach of inflating whole lung specimens and subjecting these subsequently to CT and whole lung microCT allows for an accurate matching of CT-imaging and histopathology data of exactly the same areas. Systematic application of this approach allows for a more targeted assessment of localized disease extent and more specifically can be used to investigate early mechanisms of lung diseases on a morphological and molecular level. Therefore, this technique is suitable to selectively investigate changes in the large and small airways, as well as the pulmonary arteries, veins and capillaries in relation to the disease extent in the same lung specimen. In this perspective we provide an overview of the different strategies that are currently being used, as well as how this growing field could further evolve.

## Introduction

Computed tomography (CT), positron emission tomography (PET) and histopathology are major components in the differential diagnosis and follow-up of patients with acute and chronic non-neoplastic respiratory diseases. Given the diversity in CT and histologic presentation and the inherent morphologic differences in strategies of diagnosis between neoplastic and non-neoplastic disease, we will specifically focus on non-neoplastic diseases. Especially as these tend to result in significant day-to-day challenges in the differential diagnosis.

Particularly in the field of interstitial lung diseases (ILD), there is an important diagnostic and prognostic role for CT and histopathology, with a growing emphasis on the relative weight of chest imaging as a diagnostic tool (1). Thus, the need for an invasive video-assisted or robot- assisted thoracic surgery biopsy to confirm differential diagnosis has drastically reduced. Consequently, lung biopsies are currently only recommended for those challenging cases where major discrepancies are observed between CT and clinical findings and a clear diagnosis cannot be rendered. Given this important role of imaging in the management of ILD, it is primordial to integrate the existing clinical, imaging and histopathologic data as much as possible.

Initially, assessment of remodeling and fibrosis patterns by experienced radiologists was the only way to leverage the available imaging data, but gradually and partly due to the large inter-observer variability, automated image analysis tools for characterization and quantification of CT signs and patterns have been developed of which some are on the verge of making their appearance in routine clinical care. CT can provide 3D insight into the (gross) morphological changes in the lung. However, CT patterns are often nonspecific and may change during the evolution of a disease. Importantly, CT changes may also have more than one (histo-) pathological correlate. For example, a study demonstrated that more than 90% of patients presenting with CT findings that were inconsistent with usual interstitial pneumonia demonstrated histological evidence of usual interstitial pneumonia (2). Additionally, *in vivo* resolution is rather limited with a slice thickness of 1 mm most commonly being used for routine patient CT scans due to radiation concerns. This is sufficient to investigate airways and vessels >1 mm in diameter and gross changes in the secondary pulmonary lobule and the lung parenchyma. However, the small airways, the cellular composition and specific smaller sized structures are impossible to address, although morphologic secondary effects of primary changes such as hyperinflation and air trapping may be observed.

Histopathology on the other hand provides the advantage of a detailed analysis of the morphologic and cellular changes within the lung, whereby structures in the micrometer range can be readily resolved. However, (histo-) pathology is limited due to relatively small tissue samples and only allows for a 2D investigation. This can lead to discordance between initial diagnosis made on transbronchial cryobiopsy or surgical lung biopsy and the final diagnosis made in explant lung specimens at the time of transplantation (15–22 and 12.4% respectively) (3, 4). In addition, the lung is considered quite fragile, making it vulnerable for deformation and distortion during the preparation resulting in histologic artifacts potentially hampering microscopic examination.

As, especially non-neoplastic pulmonary diseases, show a great spatial heterogeneity in disease activity and progression, targeted sampling is essential. It is reasonable to assume that often important patterns/changes in the lung can be overlooked due to sampling bias or sectioning artifacts upon histologic examination.

Proof of concept data of the huge potential of microCT, a tool designed to combine the 3D aspect of CT with almost full histological resolution, to investigate human lung (disease) was provided in 2005. However, at the time, the authors were still struggling to obtain sufficient contrast between air and tissue (5). A subsequent porcine study by Litzlbauer et al. (6) extensively validated the use of microCT with histology, showing great promise in measuring morphological changes such as alveolar surface density and mean linear intercept. Therefore, we and others have further optimized these protocols for *ex-vivo* scanning of human lung tissue to bridge the gap between radiology and histopathology, and thus between macroscopy and near-cellular resolution. In this article, we describe a unique approach of air-inflating whole human lung/lobe explants and subjecting these to a wide spectrum of investigations. We propose to use this macro to micro approach leveraging CT and microCT to bridge the gap between radiologic data and histopathology with illustrative examples. Further, we propose to use this approach to selectively investigate changes in the large and small airways, the interstitial compartment as well as the pulmonary arteries, veins and capillaries in relation to the disease extent in the same lung specimen. In addition, other promising tools are discussed in more detail including their potential applications. We acknowledge that a plethora of techniques has also been investigated in animal studies, which can readily be leveraged to combine imaging data with mechanistical insights (7); however, we deliberately focus on studies in humans that can be implemented more easily into routine clinical practice.

## Methodology of Lung Processing

Obtaining sufficient contrast between tissue and air is of major importance in the imaging of lung explants. Therefore, immediately following surgical resection, the lung is collected and cannulated via the main stem bronchus. Applying water-controlled air pressure, inflating the lung with 30 cm of water pressure and subsequently lowering the pressure to 10 cm of water pressure allows for an even and uniform recruitment of the lung specimen. The lungs are then mounted in a styrofoam box and frozen in liquid nitrogen vapors. At this moment, the lungs can be safely stored at−80°C until further use. *Ex vivo* CT scanning can be used to correlate with the last available *in vivo* CT, which already allows a higher spatial resolution due to higher radiation doses that can be used. MicroCT scanning of entire lung specimens is more complicated and a dedicated device is needed, where large samples can be mounted while keeping the lung frozen solid inside a styrofoam box. Depending on the size of the specimen, a resolution of 90–150 micron is feasible, thereby increasing the resolution 6–8 fold compared to conventional *in vivo* CT imaging.

After CT and microCT imaging, which can be used to identify structures of interest, the lung is cut in even slices using a band saw and smaller sized samples with a diameter typically between 12 and 22 mm can be extracted with a core bore or power drill depending on the degree of fibrosis within the samples. These samples can subsequently be re-scanned with high-resolution microCT preferably while keeping the sample frozen (8), where a further increase in resolution can be achieved given the smaller size of the respective specimen, typically up to 5–15 microns.

**Figure 1:**
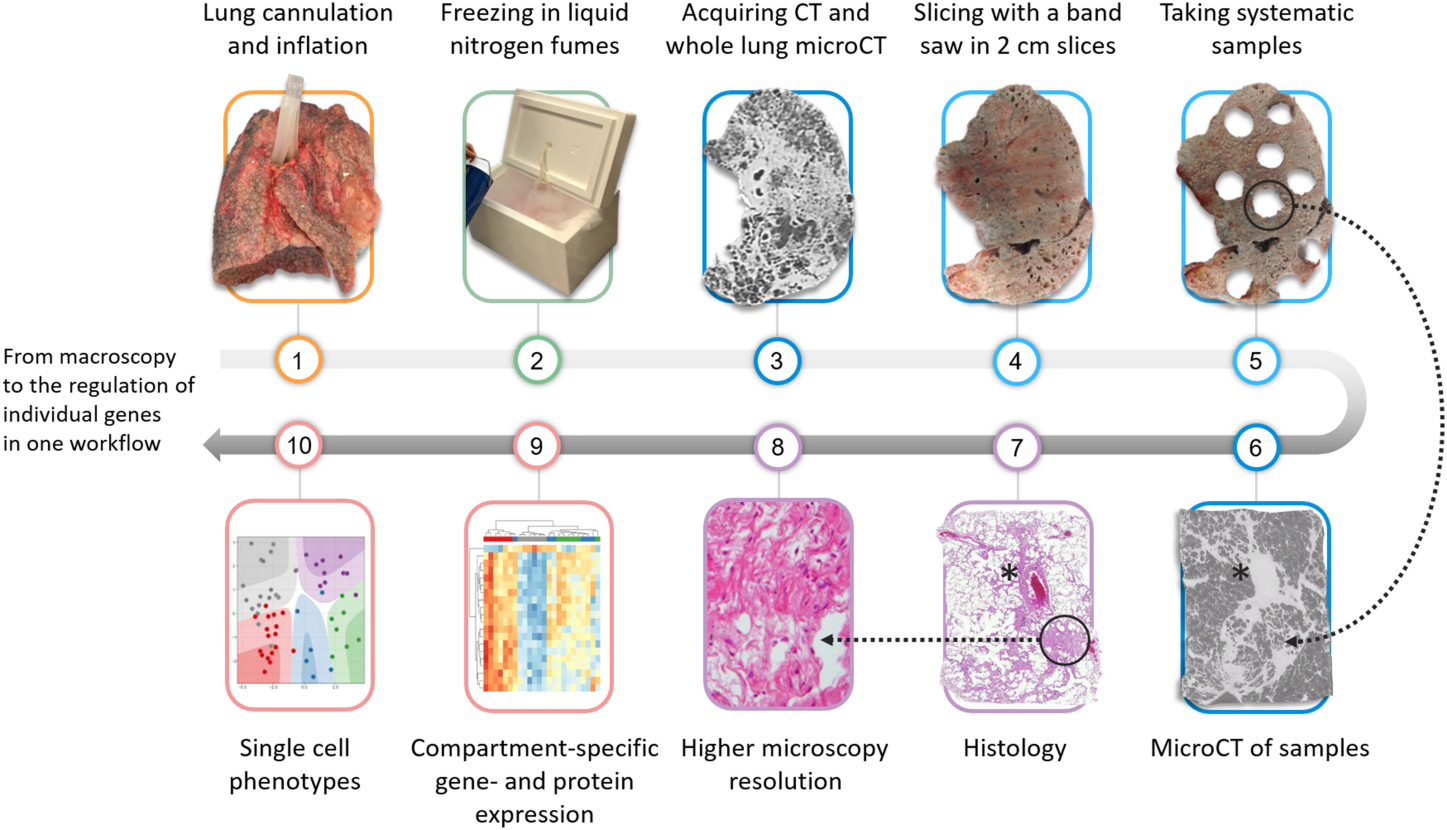
Overview of the proposed protocol that can be used to investigate the lung from macroscopy to microscopical ultrastructure. The lung is firstly cannulated via the main stem bronchus and inflated using a compressed air source and subsequently fixed in liquid nitrogen fumes. Following an *ex-vivo* CT and microCT, the lung is sliced in 2 cm slices and samples are systematically extracted using a core bore or power drill. Pre-selected or randomly selected samples can be scanned with microCT to further improve the spatial resolution. Further validation can be performed with histopathology. More downstream possible molecular analysis includes compartment-specific gene expression analysis or single nuclear analysis which can be used to elucidate specific pathways in pathological areas. *blood vessel.

Subsequently, the lung can be further processed for histopathologic and molecular assessment, e.g., by formalin fixation and dehydration of samples. Our workflow is further illustrated in Figure 1. The quality of standard histology is not severely impaired by the prior process of freezing and scanning the lung. Although minor freezing artifacts can be observed, the quality of the histology is much better compared to frozen sections, explained by the fact that the tissue is never in direct contact with ice-cold solutions such as glutaraldehyde. It is very important to emphasize that this process of freezing the lung *in toto* and using microCT also has limited negative impact on further downstream molecular analysis illustrated by e.g., similar gene expression signatures in scanned vs. non-scanned specimens (8). Also bulk RNA-sequencing (9, 10), immunohistochemistry (11), and microbiome sequencing (Einarsson et al. accepted for publication) have already been performed, making ours a valid tool for more molecular-based research.

## CT and microCT of Explant Lungs

Leveraging CT and whole lung microCT, an easier separation of the lung into morphologically inconspicuous, moderate and severely diseased areas can be made, that can be specifically sampled using the targeted sampling protocol described above. The healthy appearing areas as evident from the imaging can be used as a proxy of early disease. This is valuable given the temporal heterogeneity in non-neoplastic lung diseases, since genuine specimens with early disease features for research purposes are difficult to obtain. There is certainly some ground truth in such a hypothesis as distinct morphological and molecular processes have been demonstrated in minimally affected vs. severely affected regions (9, 10).

**Figure 2:**
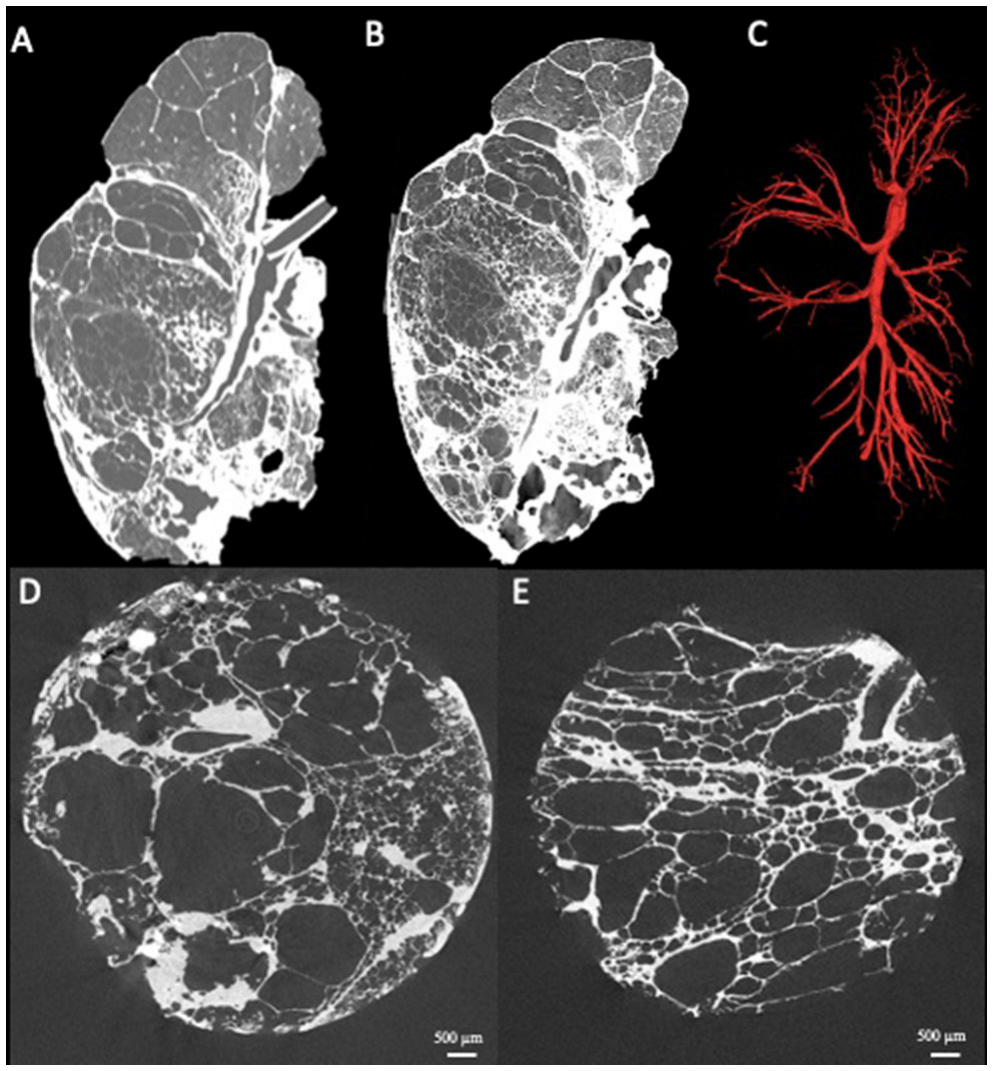
Presentation of a case where clinical routine meets research. Patient with unilateral congenital emphysema that underwent pneumonectomy. The *ex-vivo* clinical CT scan shows severe emphysematous destruction **(A)** with some fibrosis in the lower lung lobe. The whole lung microCT shows greatly improved details providing near alveolar resolution **(B)**. Airway segmentation of the whole lung by microCT demonstrating the simplification of the airway tree characterized by a low number of visible airways and remarkable long airway segments without airway branching **(C)**. MicroCT imaging of extracted lung specimens showing the severe emphysematous destruction in this lung **(D,E)**.

Next to pattern and structure analysis, it is also possible to visually identify and segment the entire bronchial tree from the main stem bronchus until the last branch of conducting airways (i.e., terminal bronchioles). This approach can therefore be used to investigate airway abnormalities like airway collapse, airway obstruction or bronchiectasis on a whole lung scale. In addition quantitative data such as the numbers of airways per generation, airway diameters and segment lengths can be generated as has been described already in COPD (12), graft-vs.-host-disease post-allogenic stem cell transplantation (13), lymphangioleiomyomatosis (11) and physiologic aging (14). The use of a contrast agent, for example osmium staining, also allows for the investigation of (micro-)vascular structures, making the estimation of vascular volumes relative to the total tissue volume possible (15). Specifically in rare pulmonary diseases, whole lung microCT can bridge the gap between research and clinical routine as exemplified in Figure 2, where a representative *ex-vivo* CT scan (Figure 2A), whole lung microCT scan (Figure 2B) and the segmented airway tree based on whole lung microCT (Figure 2C) are shown from a patient who underwent pneumonectomy for unilateral congenital emphysema (Figures 2D,E). The airway segmentation shows an aberrant airway bifurcation pattern with a marked decrease in the number of visible airways. Remarkably, there are large airway segments without notable airway branching; especially airway segments in severely emphysematous areas show a lack of airway bifurcations (Figure 2C).

## Correlation with Histopathology

Numerous insights can be generated by high-resolution imaging, however the correlation between imaging and histopathology is often difficult, given the inherent differences in spatial resolution. While high-resolution imaging data allows for the investigation of morphological changes, it only provides black and white images and changes at the cellular level or minimal density differences cannot be resolved, making routine pathology still an indispensable tool for both research and routine clinical purposes. Some attempts have, however, been made to correlate imaging with histopathology data, especially with the recent introduction of synchrotron imaging, an extremely powerful source of X-rays, of paraffin embedded samples where sub-stacks of images with (sub)micrometer resolution can be created. Hierarchical phase contrast imaging is another application of synchrotron imaging, where specialized sample preparation and equipment enable the scanning of entire lungs, but also other organs, with a resolution of 25 micron. Furthermore, this technology allows the acquisition of sub-scans with a resolution of 2.5 micron due to the high X-ray photon flux and coherence achieved at modern fourth-generation synchrotron sources (16). This technique has been employed by Ackermann and colleagues to reveal microvascular changes (i.e., broncho-pulmonary shunting) in the bronchial circulation of COVID-19 patients (17). Synchrotron imaging of paraffin embedded samples on the other hand has been used by another group to study morphological changes in the vascularity in pulmonary vascular diseases. Westroo and colleagues leveraged synchrotron imaging to demonstrate a previously unappreciated plexiform lesion heterogeneity, the pathological hallmark of (idiopathic) pulmonary hypertension (18). The same group also used synchrotron-based phase-contrast microCT in combination with vascular dye injection to investigate the vascular morphology in alveolar capillary dysplasia with misaligned pulmonary veins, with vascular dye injection being of significant help in the analysis and further segmentation of the vascular structures (19). A 3D approach for virtual histology and histopathology based on multi-scale phase contrast x-ray tomography has also been applied to punches of tissue samples with a maximum cross section of 8 mm derived from COVID-19 victims, which allows the segmentation of individual cells with a minimal resolution of 167 nm (20). Lastly, a 36 M-pixel synchrotron radiation microCT has been successfully used to study the secondary pulmonary lobule from a large human lung specimen (21).

However, this approach is not readily available due to the specific nature of the required equipment for both acquisition and analysis, the need for extensive experience with the interpretation of the images and is therefore not (yet) ready for high-throughput screening.

## microCT Imaging as a Scouting Tool

Histopathologic analysis remains the only reliable option for identifying cellular and morphologic patterns keeping the limitation of small tissue samples and the lack of 3D insight in mind. Serial sectioning of paraffin blocks has been used to extensively demonstrate the correlation between microCT and histology (14), however the process of aligning, matching and cutting the samples is tedious and time-consuming. As an alternative, microCT scanning of paraffin blocks or microCT as a scouting tool have been proposed (22). This can especially be interesting for retrospective studies, in situations where no whole organ is available or when detailed and fast histopathologic assessment is essential for routine patient care such as surgical lung biopsies in the context of a possible interstitial lung disease or suspicion of neoplasms in a resected lung specimen. Interesting and relevant regions can be selected for further sectioning based on the microCT selection which enables time-efficient preparation of conventional histological sections (22). In that aspect, it is also of interest that a novel X-ray microtomosynthesis of unstained pathology tissue samples has also been proposed. Its unique design maximizes the photon flux density through the sample as no rotation is required and therefore samples can be scanned closer to the source resulting in higher resolution (23).

In addition to its potential as a scouting tool, X-ray imaging can also assist in generating a 3D overview of morphological changes in the lung structure. Jones et al. leveraged microCT of paraffin embedded samples to investigate the 3D morphology of fibroblast foci, a key histologic feature of active fibroproliferation in the context of interstitial lung disease. In contrast to the general belief at that time, the fibroblast foci in the lungs of IPF patients were not interconnected and displayed a wide plasticity (24). Wells et al. also used microCT, histopathology, and immunohistochemistry to investigate necrotic granuloma, a characteristic feature of tuberculosis and showed that necrotic granulomas exhibit more complex shapes than anticipated, including cylindrical, branched morphologies that are connected to the airways and shaped by the bronchi (25).

The 3D overview provided by microCT is not only helpful to investigate morphologic changes but can also be used to investigate structural changes in the airways. Mcdonough et al. were the first to employ microCT to quantify the size and number of terminal bronchioles, the last generation of conducting airways before the respiratory bronchioles and alveoli, in tissue samples removed from explanted lungs from COPD patients. Terminal bronchioles can be visually identified on microCT imaging by the loss of the airway wall and appearance of alveolar buds and are of considerable importance in chronic non-neoplastic lung diseases. A significant decrease in the number and size of terminal bronchioles in end-stage COPD lungs was found compared to controls. Given that repeated sampling of the same lung was possible and given that the tissue volume of the sample could be determined, it not only allowed the assessment of the number of terminal bronchioles in the sample, but by carefully measuring the lung volume, these numbers could be extrapolated to the entire lung. This approach provided quantitative data of the actual small airway involvement in COPD for the first time, something that was always assumed but never conclusively demonstrated in a quantitative way (26). Later on, this approach has also been successfully leveraged to demonstrate small airway involvement in post lung transplantation rejection, cystic fibrosis and idiopathic pulmonary fibrosis (27–30). Over the last years, this approach has been further refined to allow scanning of the samples in frozen condition, making destructive dehydration which was applied at first, unnecessary. In addition, an improvement in the analysis tools has allowed the researchers to extract additional quantitative parameters such as the number of alveolar attachments, the thickness of the airway wall, the circularity of the lumen, but also size and diameter of the preterminal bronchioles (31).

## Discussion

Given the above evidence, it is clear that microCT can serve as a complementary, and in the future perhaps even an indispensable tool for routine pathology (32). Although significant technical advances have been made, there is still some work to be done. Indeed, specific microCT devices have already been optimized for 3D imaging of non-stained soft tissue at a resolution of 5–10 micron. These scans could be implemented in routine care and could assist in histology-guided identification of a range of tissue structures and diagnostically relevant histologic criteria and moreover may allow easy quantification of relevant measures such as for example tissue thickness (33). It is our belief that similar advances could also assist in improving the differential diagnosis, particularly in the field of non-neoplastic diseases. However, these approaches could also be leveraged to better understand neoplastic lung diseases such as micro-metastasis, the role of the tumor micro-environment and the role of the vasculature in neoplastic lung disease. Our approach also allows for structurally targeted molecular investigation with both fresh frozen–or formalin fixed tissue being available after non-destructive imaging, allowing panel-based gene expression analysis or broad next-generation sequencing approaches (34). Additionally, microorganisms such as bacteria from biofilms can be recultivated from native flash frozen tissue and used in *in-vitro* assays.

Applying microCT or synchrotron imaging directly to living patients remains an elusive dream, yet a recent study applied propagation-based phase-contrast CT on a human-scale chest phantom prepped with an inflated fresh porcine lung. The authors demonstrated that a resolution of 100 micron could be obtained in a limited local area of interest with significant less radiation than used in conventional CT scanning (35), indicating the possibilities of *in vivo* high resolution scanning. This indicates that we could dream big and hope to apply similar high-resolution imaging *in vivo*, making it perhaps even possible to avoid invasive biopsies. Caution is however needed as rigorous interpretation of the, often immense, imaging data is needed, which could cause a new wave for artificial intelligence based analysis of microCT scans, similar to the current wave of research in the use of artificial intelligence analysis of conventional CT images (36).

Therefore, the imaging-based approaches to close the gap from macroscopy to ultrastructure seems a safe and trustworthy option, which can be relatively fast implemented in the routine care of patients suffering from acute or chronic lung disease and which can facilitate the differential diagnosis.

## Data Availability Statement

The original contributions presented in the study are included in the article/supplementary material, further inquiries can be directed to the corresponding author.

## Ethics Statement

The studies involving human participants were reviewed and approved by University Hospital of Antwerp Ethical committee. The patients/participants provided their written informed consent to participate in this study.

## Author Contributions

SV, PB, CW, MA, JH, TL, JK-V-E, and DJ: responsible for conception and design of the study. EP, MK, AS, HE, TW, AH, FL, SD, PP, VV, and PV: wrote sections of the manuscript. All authors contribute to sample preparation, processing, contributed to manuscript revision, read, and approved the submitted version.

## Funding

The grants of the European Research Council (ERC); European Consolidator Grant, XHale to DJ (Ref. No. 771883).

## Conflict of Interest

The authors declare that the research was conducted in the absence of any commercial or financial relationships that could be construed as a potential conflict of interest.

## Publisher's Note

All claims expressed in this article are solely those of the authors and do not necessarily represent those of their affiliated organizations, or those of the publisher, the editors and the reviewers. Any product that may be evaluated in this article, or claim that may be made by its manufacturer, is not guaranteed or endorsed by the publisher.
